# Development and Evaluation of a Nurse Competency Assessment System for Public Health Emergencies

**DOI:** 10.1155/nrp/5873181

**Published:** 2026-04-22

**Authors:** Jingbo Jia, Cailing Xu, Yi Ren, Chao Wang, Yang Yang, Wenxin Zhang

**Affiliations:** ^1^ Tuberculosis Department, Tianjin Haihe Hospital, Tianjin, China, haiheyiyuan.cn; ^2^ TCM Key Research Laboratory for Infectious Disease Prevention for State Administration of Traditional Chinese Medicine, Tianjin, China; ^3^ Digestive System Department, Tianjin Haihe Hospital, Tianjin, China, haiheyiyuan.cn; ^4^ Nursing Department, Tianjin Haihe Hospital, Tianjin, China, haiheyiyuan.cn; ^5^ Fever Clinics, Tianjin Haihe Hospital, Tianjin, China, haiheyiyuan.cn; ^6^ Infectious Diseases Department, Tianjin Haihe Hospital, Tianjin, China, haiheyiyuan.cn; ^7^ Cardiac and Thoracic Surgery Nursing Unit, Tianjin Haihe Hospital, Tianjin, China, haiheyiyuan.cn

**Keywords:** Delphi method, designated hospitals, evaluation system, nurse competencies, public health emergencies

## Abstract

**Objective:**

This study aimed to construct a competency assessment system for nurses working in designated hospitals for public health emergencies, specifically major outbreaks of new respiratory infectious diseases, and to evaluate its usability in order to provide a standardized tool for assessing the skills of nurses caring for patients with such infectious diseases.

**Methods:**

The competency assessment index system for nurses in designated hospitals was developed through literature analysis, behavioral interviews, and the Delphi expert consultation method. The weights of the indicators were calculated using the Analytic Hierarchy Process (AHP), a structured technique for organizing and analyzing complex decisions. Subsequently, a satisfaction survey was conducted with 80 nurses from a designated hospital in Tianjin to evaluate the system’s usability.

**Results:**

The effective response rates of the expert consultation questionnaires for two rounds were 95% and 100%, respectively. The expert authority coefficient was 0.902, and Kendall’s coefficient of concordance (W) ranged between 0.135 and 0.198 (all *p* < 0.05), indicating significant agreement among experts. The final evaluation system included 4 first‐level indicators, 13 second‐level indicators, and 56 third‐level indicators. Cronbach’s alpha was 0.990, the S‐CVI/Ave was 0.996, and the I‐CVIs ranged from 0.900 to 1.000. The satisfaction score among nurses was 18.69 ± 1.19 points on a 20‐point scale.

**Conclusion:**

The competency assessment system for nurses in designated hospitals for PHEs developed in this study demonstrates strong scientific validity, reliability, and practical value. It provides a scientific basis for the selection, training, and assessment of nurses in designated hospitals for PHEs.

## 1. Introduction

The increasing frequency and severity of public health emergencies (PHEs), particularly emerging infectious disease outbreaks, present a critical test for global health security [1]. International frameworks, such as the World Health Organization’s (WHO) Health Emergency and Disaster Risk Management (Health‐EDRM) Framework [2, 3] and the International Council of Nurses’ (ICN) Disaster Nursing Competencies, provide essential strategic direction for strengthening nursing workforce resilience [4]. These guidelines universally underscore the necessity of a competent nursing workforce as the frontline defenders during crises. However, the operationalization of these global standards necessitates deliberate localization because the structure, resources, protocols, and challenges of healthcare systems vary significantly across national and institutional contexts, creating a pivotal gap between international principles and actionable, context‐specific assessment tools [5–7]. While international guidelines provide a broad framework, the need for localization is particularly acute within specialized clinical settings such as designated hospitals for public health emergencies (PHEs), which form the core of the response architecture in many health systems, including China [8]. During outbreaks like SARS and COVID‐19, these hospitals operate under uniquely intense conditions, characterized by rapid patient surges, strict zoning protocols with multiple tiers, high‐risk aerosol‐generating procedures, and prolonged staff deployment in isolation environments [9–11]. Nurses in these settings face distinct competency demands that extend beyond general disaster nursing principles; these demands integrate advanced infection prevention and control (IPC) within complex engineering controls and critical care skills for deteriorating patients in isolation wards. They also require psychological resilience or coping mechanisms for sustained high‐risk exposure, as well as specific communication skills with patients and families under visitation restrictions [12–14]. While international frameworks set the vision, a “one‐size‐fits‐all” competency assessment fails to capture these context‐specific demands, leading to gaps in practical application [15]. Existing competency frameworks often fail to address the specific demands of infectious disease emergencies, such as high transmission risk, the need for rapid intervention, and operational uncertainty [16]. A system tailored for general hospital emergency departments or community settings may not validly measure the proficiency required to safely don and doff personal protective equipment in a negative‐pressure ward, manage ethical dilemmas of resource allocation in a dedicated outbreak unit, or lead a team in a newly activated containment zone [17].

Therefore, developing a localized assessment system is not a departure from international guidelines but rather an essential implementation of those guidelines [18]. It translates broad competency domains into measurable, behaviorally anchored indicators. These indicators reflect the real‐world workflows, technological infrastructure, and institutional policies of designated infectious disease hospitals [19].

This study aims to address this urgent need by establishing a nurse competency evaluation system for a designated hospital for PHEs in response to sudden respiratory infectious disease outbreaks, based on the iceberg model [20] and the ICN competency domains as the theoretical framework. The study also performs preliminary validation to provide a scientifically rigorous and practical assessment tool. This tool will evaluate and support the development of specialized capabilities required for effective pandemic response, ultimately enhancing the resilience of healthcare systems and aligning with global health security objectives.

Ethical approval: This study was conducted in accordance with the Declaration of Helsinki and was reviewed and approved by the Institutional Review Board or Ethics Committee of Tianjin Haihe Hospital (Approval No. 2021HHKT‐020). Written informed consent was obtained from all participants involved in the study.

## 2. Methods

### 2.1. Building a Competency Evaluation System for Nurses in a Designated Hospital for PHEs

#### 2.1.1. Establishment of the Research Team

We formed a research team to evaluate nursing competency in a designated hospital for PHEs. The team includes one vice president in charge of nursing, one director of the nursing department, one head of the human resources department, one head of the education and research department, and five nursing graduate students who have received training in evidence‐based methodology and participated in emergency public health response tasks. Team members are responsible for policy guidance, performing literature searches, constructing the item pool, drafting outlines, conducting interviews, determining the initial draft of the indicator system, selecting consulting experts, carrying out two rounds of expert consultations, revising indicators, and performing statistical analysis. The research team established a regular meeting schedule; all members attend meetings punctually to ensure timely monitoring of research quality.

#### 2.1.2. Literature Search and Organization

We searched literature using keywords such as “nursing,” “nurse,” “competency,” “competency characteristics,” “core competencies,” “job competency,” and “evaluation system” in databases including CNKI, Wanfang Medical Database, VIP, China Biomedical Literature Database, PubMed, Web of Science, Embase, and CINAHL. The search covered the period from January 2011 to May 2021. Relevant literature was gathered, and representative studies were selected based on pre‐established criteria. The literature was then organized and analyzed through filtering, deduplication, and conceptual merging of competency items. This process referenced the iceberg model [20] and ICN competencies [4] and defined relevant concepts based on the current work characteristics of nursing staff in a designated hospital for PHEs, initially constructing the item pool for the competency evaluation system of nurses in a designated hospital for PHEs.

#### 2.1.3. In‐Depth Interviews

Interview participants and sample characteristics: To ensure the representativeness of the competency model, a purposive sampling strategy was employed. We selected 20 registered nurses from a designated hospital for PHEs. The sample was stratified into two groups based on their supervisors’ performance evaluations over the past 2 years. The high‐performance group (*n* = 10) consisted of nurses consistently rated as “excellent.” The average‐performance group (*n* = 10) included nurses rated as “competent.” All participants had at least 6 months of direct clinical experience in designated hospitals during the pandemic. The sample included nurses from different departments (e.g., emergency, ICU, isolation wards) with 2 to 15 years of experience to capture a breadth of perspectives.

Interview protocol and data collection: We conducted semistructured, in‐depth interviews using the Critical Incident Technique (CIT), a method that involves collecting detailed accounts of significant events to explore behaviors and competencies. A double‐blind procedure was followed, in which neither the interviewer nor the interviewee was aware of the participants’ performance grouping, to minimize bias. The interview guide centered on the following core question: “Please recall one‐to‐three of the most critical or memorable incidents you experienced while working in the designated hospital for PHEs: these could be situations you handled particularly well or found especially challenging. Please describe in detail what happened, what you did, what you were thinking at the time, and what the outcome was.” Probes were used to elicit detailed descriptions of actions, thoughts, and feelings (e.g., “What was your specific role in that situation?”; “Why did you choose that approach?”). All interviews were audio‐recorded with the interviewee’s consent and transcribed verbatim within 20 to 48 h after the interview.

Data analysis and item generation: Interview transcripts were analyzed using thematic analysis, following established procedures, to identify competency characteristics. The process involved the following steps. Two researchers independently read the transcripts and coded salient behavioral statements (e.g., “I immediately coordinated with the doctor to check the ventilator settings”). Codes were then compared and clustered into preliminary competency themes, such as emergency equipment management and interprofessional collaboration. The emerging themes were continuously compared against the original incidents and discussed among the research team members. Discrepancies were resolved through consensus among the team. Each consolidated competency theme was translated into a measurable behavioral indicator based on predefined criteria to form the item pool. For instance, the theme of interprofessional collaboration informed items such as “Ability to communicate with people at all levels and departments.”

#### 2.1.4. Expert Consultation

Selection of consulting experts: A two‐round Delphi method was employed to build consensus. The expert panel was selected through a combination of purposive and snowball sampling to ensure both expertise and diversity.

Inclusion criteria for experts were: ① Professional authority: Held a senior professional title (e.g., chief nurse, professor, and senior physician) and possessed at least 10 years of hands‐on and managerial experience in the field of respiratory infectious diseases, emergency departments, ICUs, or public health management. ② Expertise and reputation: Recognized as a high‐level expert in the fields of respiratory infectious disease management and PHEs, often evidenced by leadership roles in relevant committees or past involvement in policy development. ③ Educational background: Held a bachelor’s degree or higher, with a preference for those with advanced degrees (master’s or PhD) in nursing, public health, or medicine. ④ Geographical representation: Experts were drawn from diverse regions to mitigate regional bias, including from tertiary hospitals, centers for disease control, and university settings. ⑤ Commitment and availability: Demonstrated willingness and guaranteed availability to actively participate in the entire two‐round consultation process.

Initially, 17 experts were invited to participate to account for potential attrition. Prior to their participation, each invited expert received a detailed information sheet via email, which outlined the study’s purpose, procedures, estimated time commitment, and the voluntary and anonymous nature of their participation. They were informed that by proceeding to complete and return the online questionnaire, they were providing their implied informed consent. This consent procedure was explicitly approved by the ethics committee. In the first round, 15 experts completed the questionnaire (response rate: 88.23%). All 15 experts from the first round subsequently participated in the second round (response rate: 100%), resulting in a final panel of 15 experts who completed both rounds for data analysis. The high response rates in both rounds underscore the panel’s strong engagement and the perceived importance of the study. Fifteen experts from East China, West China, South China, Northeast China, and the Beijing–Tianjin region participated in the consultation, with more details about the experts in Table 1.

**TABLE 1:** General data of experts.

No.	Gender	Age	Educational background	Technical title	Research field	Professional years
1	Female	49	Undergraduate	Chief superintendent nurse	Infectious disease care and management	30
2	Female	45	Master	Co‐chief superintendent nurse	Public health emergency care management	25
3	Female	56	Undergraduate	Chief superintendent nurse	Care for infectious diseases	25
4	Female	51	Undergraduate	Chief superintendent nurse	Respiratory disease care	32
5	Female	52	Undergraduate	Chief superintendent nurse	Neurological disease care	29
6	Female	55	Undergraduate	Chief superintendent nurse	Respiratory disease care	32
7	Female	67	Undergraduate	Chief superintendent nurse	Nursing supervision	47
8	Female	58	Master	Chief superintendent nurse	Critical care and management of acute and severe illness	30
9	Female	53	Undergraduate	Chief superintendent nurse	Critical care	34
10	Female	56	Undergraduate	Chief superintendent nurse	Nursing supervision	10
11	Female	61	Undergraduate	Chief superintendent nurse	Critical care and nursing management	41
12	Female	56	Undergraduate	Chief superintendent nurse	Respiratory disease care	23
13	Female	53	Undergraduate	Chief superintendent nurse	Respiratory disease care	31
14	Female	48	Undergraduate	Chief superintendent nurse	Respiratory disease care	24
15	Female	53	Undergraduate	Chief superintendent nurse	Respiratory and critical care	19

Definition of consensus: The consensus criteria for each indicator in each round of the Delphi consultation were predefined as follows:

Mean score: An indicator was retained if its mean importance score was ≥ 4.0 (on a 5‐point Likert scale).

Coefficient of variation (CV): A CV ≤ 0.25 was considered to indicate a good level of agreement among experts.

Kendall’s coefficient of concordance (W): A statistically significant Kendall’s W (*p* < 0.05) for each dimension (e.g., all indicators under a primary indicator) was considered to indicate good overall coordination of expert opinions [21].

The final decision on the inclusion, exclusion, or modification of an indicator was based on a comprehensive consideration of the above quantitative criteria together with the qualitative suggestions provided by the experts.

Management of inter‐round feedback: The processing of expert suggestions and the development of the subsequent round questionnaire were conducted as follows: After each round, all quantitative data (mean scores, standard deviations, and coefficients of variation) and qualitative suggestions were systematically collated.

The research team, consisting of two graduate student researchers and the principal investigator, reviewed all qualitative suggestions. Each suggestion was discussed in relation to the quantitative data and the specific objectives of the study. A consensus decision within the research team was required to implement any change.

The principles for modifications were as follows: deletion was applied when an indicator received a low mean score (< 3.5) and/or experts consistently indicated that the indicator was irrelevant or overlapping.

Merging: Indicators were merged if three or more experts indicated significant conceptual overlap between them.

Modification: Indicator wording was modified if suggestions aimed to improve clarity, precision, or scope without altering the core concept.

For the second round, the revised list of indicators was presented to the expert panel. Experts received a summary report showing the following:

a. Their own first‐round ratings;
b. The group’s statistical results (mean, CV) for each indicator; and
c. A concise list of all suggestions received and the corresponding modifications made by the research team (e.g., “Indicator A was merged into Indicator B based on suggestions from three experts”).

Experts were then asked to rerate the revised set of indicators based on this feedback.

#### 2.1.5. Implementation of the Consultation

From October to December 2021, the consultation questionnaire was sent to experts via email and WeChat. Prior to each survey, sufficient communication was conducted with experts to ensure understanding and engagement. Two rounds of questionnaire surveys were conducted.

After collecting the first round of questionnaires, data were processed and analyzed under the guidance of a statistician. Modifications to the indicators were made based on the selection criteria and expert opinions, forming a second round of consultation questionnaires, which were then sent to experts for the second round.

Data from the second round were analyzed, and indicators were further refined. Experts were then asked to evaluate the content validity of all items.

#### 2.1.6. Analytic Hierarchy Process (AHP) for Weight Calculation

Following the establishment of the final competency indicator system via the Delphi method, the AHP was employed to determine the relative weights of indicators at all levels.

The structured AHP questionnaires were distributed to the same expert panel. Experts were asked to compare the relative importance of indicators within the same hierarchical level (e.g., comparing the four primary indicators against each other), using the standard 1–9 scale of relative importance.

For each expert’s comparison matrix, the local priority vector (weight) for each indicator was calculated using the eigenvalue method. The consistency of each matrix was rigorously checked by calculating the consistency ratio (CR). A matrix was deemed acceptable only if CR < 0.10; otherwise, the expert was requested to review and revise their judgments.

To derive a group decision, the acceptable individual judgment matrices were aggregated using the geometric mean method to form a single comparison matrix for each level. The final local weights for each level were calculated from the aggregated matrices.

Subsequently, the global (or combined) weight for each tertiary indicator was computed by multiplying its local weight by the local weights of all its parent indicators in the hierarchy.

The sensitivity of the final ranking of indicators to changes in the primary‐level weights was tested to assess the robustness of the AHP model [22].

### 2.2. Usability Evaluation of the Competency Evaluation System for Nurses in Designated Hospitals for PHEs

#### 2.2.1. Survey Subjects

We used convenience sampling to select 80 frontline nurses who participated in clinical nursing work for newly emerging respiratory infectious diseases in designated hospitals in Tianjin for the survey. Prior to data collection, the principal investigator approached potential participants in person. Each nurse was provided with a detailed written information sheet and had the opportunity to verbally discuss the study’s aims, risks, and benefits. They were assured that participation was entirely voluntary, refusal to participate did not result in any penalty, and all data would be anonymized. All 80 nurses provided written informed consent before receiving the questionnaire.

The inclusion criteria were as follows: ① Obtained a nursing practice certificate and registered at this medical institution; ② Engaged in nursing work in this city for 1 year or more; ③ Participated in nursing work for designated hospitals for PHEs for 14 days or more; and ④ Voluntarily participated in this study and signed informed consent.

The exclusion criteria were as follows: ① Unable to participate in this study due to studying, training, attending meetings, or taking leave; ② Questionnaires exhibiting identical or repetitive response patterns; and ③ Questionnaires with a missing response rate exceeding 10%.

#### 2.2.2. Survey Tools

The survey tools consisted of two parts: A general information form and a satisfaction questionnaire about the competency evaluation system for nurses in designated hospitals for PHEs. The general information form included gender, age, marital status, highest education level, major, professional title, level, employment form, and position. The satisfaction questionnaire was self‐developed by the research team based on a literature review [23] and covered feasibility, professionalism, comprehensiveness, and necessity of application of the evaluation system. Each item used a Likert 5‐point scoring method, ranging from 1 to 5, corresponding to *very dissatisfied* to *very satisfied*, with a total score ranging from 4 to 20. A higher score indicated greater satisfaction with the evaluation system.

#### 2.2.3. Data Collection Methods and Quality Control

The survey was conducted by the researcher and another trained member of the research team. Nursing staff participating in PHE response were selected based on the inclusion and exclusion criteria. The researcher provided clear instructions on how to complete the questionnaire and addressed any questions that arose during the survey. Returned questionnaires were checked on‐site. For those with missing items, after explanation, respondents were asked to modify and complete them. Questionnaires were collected only after confirming their accuracy.

### 2.3. Statistical Methods

We used Excel 2017, SPSS 22.0, and Yaahp 10.3 for statistical analysis. Normally distributed measurement data were described using mean ± standard deviation, while non‐normally distributed measurement data were described using median and interquartile range. Count data were described using frequency and percentage. To assess the psychometric properties of the finalized competency evaluation system, we calculated both internal consistency reliability and content validity indices. Internal consistency was evaluated using Cronbach’s alpha for the entire scale. Content validity was assessed by computing the Item‐Content Validity Index (I‐CVI) and the Scale‐Content Validity Index (S‐CVI), based on previously obtained expert ratings of each item’s relevance.

## 3. Results

### 3.1. Expert Consultation Results

#### 3.1.1. Reliability of Expert Consultation Results

In the first round, 26.67% (4 experts) provided suggestions for modifications, while in the second round, 6.67% (1 expert) did. The authority scores of the experts in both rounds were 0.900, with judgment coefficients and familiarity levels being 0.967 and 0.833, respectively.

#### 3.1.2. Concentration of Expert Consultation Results

The experts′ opinions showed a high degree of concentration. As detailed in Table 2, in the second round, the mean scores for almost all retained indicators were above 4.50. This indicates a strong convergence of expert opinions toward a consensus.

**TABLE 2:** Concentration of expert opinions.

Round	Indicators	Average value	Standard deviation	Full marks frequency
First round	First‐level indicators	4.60∼5.00	0∼0.63	66.7∼100
	Secondary indicators	4.20∼5.00	0∼0.21	40.0∼100
	Third indicator	3.73∼5.00	0∼0.88	13.3∼100

Second round	First‐level indicators	4.67∼5.00	0∼0.62	73.3∼100
	Secondary indicators	4.53∼5.00	0∼0.64	60.0∼100
	Third indicator	4.20∼5.00	0∼0.83	33.3∼100

#### 3.1.3. Coordination of Expert Opinions

The coordination among experts, as measured by Kendall’s W, was high and statistically significant. For the primary indicators, the Kendall’s W values ranged from 0.150 to 0.371 (all *p* < 0.05), indicating a strong and statistically significant consensus (see Table 3). The CV for each indicator, as shown in Table 2, also confirmed a high level of agreement.

**TABLE 3:** Coordination of expert opinions and significance testing.

Round	Indicators	Coefficient of variation	Kendall coefficient	*χ* ^2^	Degree of freedom	*p*
First round	First‐level indicators	0.074	0.237	10.66	3	0.014
	Secondary indicators	0.056	0.298	53.681	12	< 0.001
	Third indicator	0.088	0.371	333.943	60	< 0.001

Second round	First‐level indicators	0.055	0.230	10.355	3	0.016
	Secondary indicators	0.062	0.150	26.924	12	0.008
	Third indicator	0.053	0.269	213.744	55	< 0.001

#### 3.1.4. Results of Expert Consultation

In the two rounds of expert consultation, four experts in the first round and one expert in the second round, respectively, provided constructive suggestions. After the first round, two tertiary indicators, including “evidence‐based capability,” were removed, and some tertiary indicators were merged; for example, “narrative nursing knowledge” was incorporated into “humanistic care and psychological nursing for public health emergencies.” Some tertiary indicators were also modified, such as changing “use and precautions of instruments and equipment related to public health emergencies” to “application and management of facilities and equipment in isolation wards.” In the second round, the primary indicator “clinical nursing practice ability” was modified to “clinical nursing practice competence.”

The final competency evaluation indicator system for nurses in designated hospitals includes four primary indicators, 13 secondary indicators, and 56 tertiary indicators. The AHP was used to calculate the weights and combined weights for each indicator, as shown in Table 4.

**TABLE 4:** Results of the second round of expert consultation on indicators.

Indicators	Importance assignment (score, mean $\overset{¯}{X}\pm S$)	Coefficient of variation	Weight	Combination weight
I speculative knowledge	5.00 ± 0.00	0.000	0.2567	—
I‐1 Basic medical knowledge of public health emergencies	4.87 ± 0.35	0.072	0.2485	0.0638
I‐1‐1 Theoretical knowledge of public health emergencies	5.00 ± 0.00	0.000	0.2077	0.0132
I‐1‐2 Diagnosis of public health emergencies	4.73 ± 0.46	0.097	0.1965	0.0125
I‐1‐3 Treatment plans for public health emergencies, etc.	4.87 ± 0.35	0.072	0.2023	0.0129
I‐1‐4 Pharmacological knowledge and usage of drugs commonly used in public health emergencies	4.8 ± 0.41	0.086	0.1994	0.0127
I‐1‐5 New progress in the study of public health emergencies	4.67 ± 0.49	0.105	0.1940	0.0124
I‐2 Emergency public health care knowledge	4.93 ± 0.26	0.052	0.2515	0.0646
I‐2‐1 Respiratory infectious disease nursing and common complications nursing	5.00 ± 0.00	0.000	1.0417	0.0081
I‐2‐2 Routine care of public health emergencies, relevant guidelines, expert consensus, etc.	5.00 ± 0.00	0.000	1.0417	0.0081
I‐2‐3 Observation of commonly used drugs and adverse reactions (including Chinese medicine and new drugs) in emergency public health diseases	4.93 ± 0.26	0.052	1.0271	0.0080
I‐2‐4 Humanistic care and psychological care for public health emergencies	5.00 ± 0.00	0.000	1.0417	0.0081
I‐2‐5 Safety management of patients with public health emergencies: such as falls, pressure injuries, suicide	5.00 ± 0.00	0.000	1.0417	0.0081
I‐2‐6 Health education for patients with public health emergencies	5.00 ± 0.00	0.000	1.0417	0.0081
I‐2‐7 Care and case analysis of severe patients with public health emergencies	5.00 ± 0.00	0.000	1.0417	0.0081
I‐2‐8 Predictive nursing and hospice care for patients with public health emergencies	4.8 ± 0.41	0.086	1.0000	0.0078
I‐3 Knowledge of prevention and control of infectious diseases in public health emergencies	5.00 ± 0.00	0.000	0.2551	0.0655
I‐3‐1 Division of functions and cleaning and disinfection of each area in the isolation ward	5.00 ± 0.00	0.000	0.3392	0.0222
I‐3‐2 Outbreak prevention and control concepts, methods and new advances in public health infection	4.87 ± 0.52	0.106	0.3304	0.0216
I‐3‐3 Public health knowledge for public information on emerging public health diseases	4.87 ± 0.35	0.072	0.3304	0.0216
I‐4 Emergency public health laws, regulations and institutional norms	4.8 ± 0.56	0.117	0.2449	0.0629
I‐4‐1 Laws and regulations related to public health emergencies	4.93 ± 0.26	0.052	0.3318	0.0209
I‐4‐2 Work system and standard procedures related to public health emergencies and isolation wards	5.00 ± 0.00	0.000	0.3365	0.0211
I‐4‐3 Hospital infection management related laws and regulations	4.93 ± 0.26	0.052	0.3318	0.0209
II Clinical nursing practice ability	5.00 ± 0.00	0.000	0.2567	—
II‐1 Emergency public health care skills	5.00 ± 0.00	0.000	0.5035	0.1292
II‐1‐1 Basic nursing techniques for public health emergencies	5.00 ± 0.00	0.000	0.1261	0.0163
II‐1‐2 Various oxygen therapy techniques	4.93 ± 0.26	0.052	0.1243	0.0161
II‐1‐3 Skills in observation, assessment, and prediction of patients with public health emergencies	5.00 ± 0.00	0.000	0.1261	0.0163
II‐1‐4 Rescue and nursing techniques for critically ill patients with public health emergencies	5.00 ± 0.00	0.000	0.1259	0.0163
II‐1‐5 Application and management of facilities and equipment in isolation ward	4.87 ± 0.52	0.106	0.1228	0.0159
II‐1‐6 New technology in care of public health emergencies	4.93 ± 0.26	0.052	0.1243	0.0161
II‐1‐7 Technology of collection, preservation, and transport of specimens of patients with public health emergencies	5.00 ± 0.00	0.000	0.1263	0.0163
II‐1‐8 Disposal of bodies of patients infected with an outbreak of public health disease	4.93 ± 0.26	0.052	0.1243	0.0161
II‐2 Outbreak public health hospital infection prevention skills	4.93 ± 0.26	0.052	0.4965	0.1274
II‐2‐1 Infection prevention and control methods and new progress of patients with public health emergencies	5.00 ± 0.00	0.000	0.1976	0.0252
II‐2‐2 Proper wearing and removal of personal protective equipment	5.00 ± 0.00	0.000	0.1686	0.0215
II‐2‐3 Emergency response methods after occupational exposure	5.00 ± 0.00	0.000	0.1976	0.0252
II‐2‐4 Methods for collection and transport of medical waste in isolation wards	4.87 ± 0.52	0.106	0.1925	0.0245
II‐2‐5 Disinfection methods for the environment, surfaces, and air in isolation wards	4.93 ± 0.26	0.052	0.2437	0.0311
III Character skills	4.80 ± 0.41	0.086	0.2467	—
III‐1 Teamwork ability	5.00 ± 0.00	0.000	0.2596	0.0615
III‐1‐1 Ability to organize and coordinate	4.73 ± 0.46	0.097	0.1620	0.0104
III‐1‐2 risk awareness	5.00 ± 0.00	0.000	0.1712	0.0110
III‐1‐3 team spirit	5.00 ± 0.00	0.000	0.1712	0.0110
III‐1‐4 Resource sharing capability	4.87 ± 0.35	0.072	0.1668	0.0107
III‐1‐5 Training and teaching capacity	4.67 ± 0.49	0.105	0.1599	0.0102
III‐1‐6 strain capacity	4.93 ± 0.26	0.052	0.1688	0.0108
III‐2 Interpersonal skills	4.80 ± 0.56	0.117	0.2492	0.0632
III‐2‐1 Ability to communicate with people at all levels and departments	4.87 ± 0.35	0.072	0.3529	0.0217
III‐2‐2 Interpersonal insight	4.73 ± 0.46	0.097	0.3428	0.0211
III‐2‐3 Daily foreign language communication skills	4.20 ± 0.68	0.161	0.3043	0.0187
III‐3 independent learning ability	4.93 ± 0.26	0.052	0.2560	0.0641
III‐3‐1 The awareness and motivation to learn new things	4.87 ± 0.35	0.072	0.3511	0.0222
III‐3‐2 Self‐planning	4.47 ± 0.83	0.187	0.3223	0.0204
III‐3‐3 self‐progression	4.53 ± 0.83	0.184	0.3266	0.0206
III‐4 Scientific research innovation ability	4.53 ± 0.64	0.141	0.2352	0.0580
III‐4‐1 Ability to design and write scientific research projects	4.20 ± 0.68	0.161	0.4773	0.0277
III‐4‐2 Ability to accurately acquire information and collect data	4.60 ± 0.63	0.137	0.5227	0.0303
IV Trait/motivation	4.67 ± 0.62	0.132	0.2399	—
IV‐1 occupational values	4.93 ± 0.26	0.052	0.3379	0.0778
IV‐1‐1 Professional dedication and social responsibility	5.00 ± 0.00	0.000	0.2518	0.0204
IV‐1‐2 Work Ethic	5.00 ± 0.00	0.000	0.2518	0.0204
IV‐1‐3 Be prudent and be alone	4.93 ± 0.26	0.052	0.2482	0.0201
IV‐1‐4 Active service awareness	4.93 ± 0.26	0.052	0.2482	0.0201
IV‐2 Physical and mental qualities	4.93 ± 0.26	0.052	0.3379	0.0811
IV‐2‐1 Good physical fitness	5.00 ± 0.00	0.000	0.3362	0.0273
IV‐2‐2 Optimism and confidence	4.87 ± 0.35	0.072	0.3275	0.0266
IV‐2‐3 Self‐emotion and psychological control ability	5.00 ± 0.00	0.000	0.3362	0.0273
IV‐3 Motivation	4.73 ± 0.46	0.097	0.3242	0.0811
IV‐3‐1 Achievement motive	4.73 ± 0.46	0.097	0.3242	0.0252
IV‐3‐2 Egemony	4.93 ± 0.26	0.052	0.3379	0.0263
IV‐3‐3 Organizational awareness	4.93 ± 0.26	0.052	0.3379	0.0263

The four primary indicators represent the foundational pillars of nursing competency in designated hospitals. These are:

Theoretical knowledge, which serves as the foundation and includes basic medical knowledge of PHEs, emergency public health care knowledge, knowledge of prevention and control of infectious diseases in PHEs, and emergency public health laws, regulations, and institutional norms.

Clinical nursing practice competence, which constitutes the core of practical execution and covers critical areas such as emergency public health care skills and outbreak public health hospital infection prevention skills.

Professional and interpersonal skills, reflecting broader responsibilities including teamwork ability, interpersonal skills, independent learning ability, and scientific research innovation ability.

Personal traits and motivation, representing intrinsic qualities that drive performance, summarized under themes like occupational values, physical and mental qualities, and motivation.

To illustrate the content coverage of the developed competency assessment system, we present selected tertiary indicators from Table 4 as examples. For instance, under the domain “I Theoretical Knowledge,” the tertiary indicator “I‐1‐1 Theoretical knowledge of public health emergencies” (score: 5.00 ± 0.00) reflects the foundational knowledge component. Similarly, under “II Clinical Nursing Practice Competence,” indicators such as “II‐1‐7 Techniques for collection, preservation, and transport of specimens of patients with public health emergencies” (score: 5.00 ± 0.00) and “II‐2‐2 Proper wearing and removal of personal protective equipment” (score: 5.00 ± 0.00) demonstrate the integration of both technical and safety competencies. These examples collectively illustrate the system’s comprehensive coverage across knowledge, skills, character, and motivational domains. Other indicators are detailed in Table 4.

#### 3.1.5. Psychometric Evaluation of the Competency System

The results of the psychometric evaluation of the competency system demonstrated strong reliability and validity. The internal consistency, as measured by Cronbach’s alpha, was 0.990, and the Cronbach’s *α* coefficients of the dimensions ranged from 0.959 to 0.985, indicating excellent reliability; see Table 5 for details. The content validity indices also showed strong results, with an S‐CVI/Ave of 0.996 and I‐CVIs ranging from 0.900 to 1.000, confirming that the content validity of this questionnaire is very good.

**TABLE 5:** The internal consistency of the dimensions.

Item	Cronbach’s *α*
Total	0.990
I speculative knowledge	0.985
II clinical nursing practice ability	0.971
III character skills	0.962
IV trait/motivation	0.959

### 3.2. Usability Evaluation Results of the Competency Evaluation System for Nurses in Designated Public Health Hospitals

A total of 80 nurses participated in the survey, including 11 males and 69 females, aged 27 to 51 years, with work experience ranging from 3 to 24 years. Among them, 27 nurses had intermediate or higher professional titles, and all 80 nurses had a bachelor’s degree or higher. The satisfaction questionnaire received a total score of 18.69 ± 1.19 (out of a maximum of 20). Given that the theoretical midpoint of the total scale is 12 [(4 + 20)/2], and a score above 16 (80% of the maximum) is commonly considered to indicate high satisfaction in similar studies, the obtained score of 18.69 (93.5% of the maximum) reflects a very high level of overall satisfaction with the developed competency evaluation system among the participants. The scores for the four dimensions were: feasibility 4.71 ± 0.52, professionalism 4.69 ± 0.62, comprehensiveness 4.63 ± 0.66, and application necessity 4.66 ± 0.61 (each out of 5). All dimension scores were well above the midpoint of 3, further confirming the positive reception.

## 4. Discussion

### 4.1. Scientific Rigor and Innovation of the Constructed System

This study developed a competency evaluation system for nurses in designated PHE hospitals through a structured process integrating literature analysis, behavioral event interviews, and a two‐round Delphi expert consultation. The high engagement (100% response rate) and the panel’s high level of expertise and authority, coupled with statistically significant consensus, affirm the robustness of the methodology and the reliability of the derived indicators.

The key innovation of this system lies in its specificity and hierarchical structure tailored for PHEs, distinguishing it from established generic nursing competency frameworks. For instance, compared to the WHO Global Competency Framework for Nurses (2020) [24] or the American Association of Colleges of Nursing (AACN) Essentials [25], which outline broad core competencies, our system drills down into PHE‐specific tertiary indicators such as II‐1‐7 Technology of collection, preservation, and transport of specimens and II‐2‐5 Disinfection methods for the environment in isolation wards. This granularity enables precise assessment of outbreak‐response capabilities.

Unlike management‐oriented frameworks like the Chinese Hospital Nurse Competency Evaluation Model [12], our system integrates Character Skills (Domain III) and Trait/Motivation (Domain IV)—including teamwork, risk awareness, resilience, and professional values—as foundational domains weighted equally with knowledge and clinical skills. This holistic design recognizes that nontechnical skills are critical for effective performance in high‐stress, resource‐constrained emergency settings [26].

The system’s architecture, organized into four primary domains, 13 secondary categories, and 56 tertiary indicators, provides a comprehensive and actionable blueprint for both assessment and targeted capacity development, filling a gap in standardized tools for PHE nursing readiness. The quantitative rigor of this blueprint is enhanced by the AHP‐derived weights assigned to each indicator. This moves the system beyond a simple checklist by establishing a clear hierarchy of competency importance. For instance, the higher combined weight of indicators within the “Clinical Nursing Practice Competence” domain (0.2567) quantitatively underscores its critical role, offering a data‐driven foundation for hospital administrators to justify and prioritize investments in related training and equipment, a significant practical improvement over many existing qualitative frameworks.

### 4.2. Implications for Nursing Education, Training, and Strategic Workforce Deployment

The constructed system transcends mere assessment; it serves as a strategic roadmap for strengthening the nursing workforce’s preparedness for crises.

For nursing education and curriculum development, the tertiary indicators can be directly translated into specialized training modules and simulation scenarios. For example, I‐2‐4 (humanistic and psychological care) and II‐1‐8 (disposal of bodies) address sensitive yet crucial areas often underrepresented in standard curricula. Integrating these competencies into preservice education and continuing professional development programs is essential for building a resilient nurse pipeline.

For in‐service training and competency assurance, the system enables gap analysis and personalized learning pathways. Hospitals can use it to identify skill deficiencies (e.g., in III‐4 Scientific research innovation ability) and design targeted training, thereby moving beyond one‐size‐fits‐all approaches to truly competency‐based education.

For workforce planning and deployment during emergencies, by profiling nurses’ competencies across domains, nursing managers can strategically mobilize personnel. A nurse excelling in III‐1‐1 (organization and coordination) and II‐1‐3 (patient observation and assessment) might be optimally deployed to a triage or team leadership role, while those with high scores in IV‐2 (physical and mental qualities) might be assigned to high‐intensity care units. This data‐driven deployment can optimize team performance and patient outcomes during patient surges in PHEs.

### 4.3. Limitations and Future Directions for Validation and Application

While this study establishes a scientifically grounded framework, we acknowledge its limitations, which chart a course for future research. Contextual Generalizability: The expert consultation and preliminary validation were conducted within a single regional context and hospital system. The homogeneity of the expert panel, though authoritative, may introduce cultural or practice bias, potentially limiting immediate applicability to diverse healthcare settings internationally. Specifically, the panel was composed entirely of senior female nursing managers from China. This demographic profile, while reflecting deep experience in managing protocols and systems, may inherently emphasize indicators related to stability, regulatory compliance, and hierarchical management structures. Consequently, the system could potentially underrepresent competencies that are highly valued in other cultural contexts, such as individual autonomy in clinical decision‐making or the perspectives of a newer generation of nurses, who might prioritize technological adaptability and different forms of interdisciplinary collaboration. This potential emphasis is an important consideration for international adaptation. Current validation is based on expert consensus and satisfaction surveys. The system lacks empirical validation in live PHE settings or through large‐scale implementation correlating assessment scores with actual job performance or patient outcomes. The indicator system, though comprehensive now, may require future updates to address emerging pathogens, technological advancements (e.g., tele‐nursing in isolation), or evolving ethical challenges.

To address these limitations, we propose the following research agenda: First, we will collaborate with designated hospitals across different regions and countries to test and adapt the system, ensuring its broader relevance and robustness. Second, we will develop the system into a digital assessment tool to facilitate real‐time competency tracking, big data analysis for workforce planning, and integration with simulation‐based training outcomes. A key feature of this digital platform could be a dynamic update mechanism designed to counter the inherent limitation of a static indicator system in the face of rapidly evolving public health threats. This mechanism, governed by an expert oversight committee, would allow for localized, evidence‐based micro‐adjustments to tertiary indicators—for instance, updating item II‐1‐6 (new technology in care) to include tele‐nursing protocols for a novel pathogen, or refining II‐2‐1 (infection prevention methods) based on new WHO or CDC guidelines. This would ensure the assessment tool remains current, contextually relevant, and capable of evolving alongside both the science of emergency response and the characteristics of the nursing workforce. Third, we will implement the system in clinical practice to conduct longitudinal studies examining its predictive validity for nurse performance, well‐being, and, ultimately, various aspects of patient care quality during actual PHEs.

## 5. Conclusion

This study developed and validated a competency assessment system for nurses responding to PHEs through a structured Delphi process. The system comprises four primary domains, 13 secondary categories, and 56 tertiary indicators relevant to emergency nursing practice.

To support implementation in clinical and managerial settings, the system can be integrated into nursing professional development programs through competency‐based training modules, periodic skills assessments, and digital evaluation tools. Its adaptable structure allows hospitals to tailor indicators and weights according to local needs, resource availability, and the type of public health threat.

While offering a structured tool for enhancing emergency preparedness among nurses, further real‐world validation across multiple settings is recommended to examine its impact on nursing performance, patient safety, and outbreak response outcomes.

## Funding

The study was funded by Tianjin Key Medical Discipline Construction Project (Grant No. TJYXZDXK‐3‐018B).

## Ethics Statement

This study was approved by Tianjin Nursing Society in China, with Ren Yi as the principal investigator. The research institution is Tianjin Haihe Hospital, China; Ethics approval number: 2021HHKT‐020; Approval date: July 14, 2021. Informed consent was obtained from all participants who agreed to participate in the study. All stages of the study were conducted in accordance with the Declaration of Helsinki.

## Consent

Informed consent was obtained from all participants.

## Conflicts of Interest

The authors declare no conflicts of interest.

## Data Availability

The authors confirm that they can provide raw data if needed.
